# Development of two instruments for assessing maternity health needs: protocol of a clinimetric study

**DOI:** 10.1186/s12884-020-03377-x

**Published:** 2020-11-17

**Authors:** Carmen Paz-Pascual, Isabel Artieta-Pinedo, Maite Espinosa, Paola Bully, ema-Q Group, Sonia Alvarez, Pilar Amorrortu, Mónica Blas, Inés Cabeza, Itziar Estalella, Ana Cristina Fernández, Marie Pierre Gagnon, Gloria Gutiérrez de Terán-Moreno, Kata Legarra, Gorane Lozano, Amaia Maquibar, David Moreno-López, Mª. Jesús Mulas, Covadonga Pérez, Angela Rodríguez, Mercedes Sáenz de Santamaría, Jesús Sánchez, Mª. José Trincado, Gema Villanueva

**Affiliations:** 1 Atención Primaria en Salud, Prevención y Enfermedades Crónicas, IIS, Biocruces Bizkaia, Osakidetza, Plaza de Cruces 12, 48903 Barakaldo, Bizkaia Spain; 2 Primary Care Midwife, Markonzaga Health Centre, Sestao, Bizkaia Spain; 3 Midwifery Training Unit of the Basque Country, Bilbao, Spain; 4 Primary Care Midwife, Zuazo Health Centre, Barakaldo, Bizkaia Spain; 5 School of Nursing, University of the Basque Country, Leioa, Bizkaia Spain; 6 Methodological and Statistical Consulting, Sopuerta, Bizkaia Spain

**Keywords:** Maternal education, E-health, Needs assessment instruments, Clinimetric properties

## Abstract

**Background:**

There is an unquestionable need to adapt health care to the needs of each woman, to foster her self-confidence and provide her with the autonomy to manage her own maternity. This involves empowering her to choose and face her model of childbirth and childcare responsibly. The range of self-management health needs tests offered by the scientific community at this stage of life is practically non-existent. In this project, we intend to develop and evaluate the validity, reliability and ease of use of two self-administered analysis instruments for: 1.- Needs of women preparing for childbirth and 2.- Identification of alarm symptoms in the puerperium.

**Methods:**

This is a descriptive study of the clinimetric characteristics and usability of two developed self-applied digital instruments for measuring needs in childbirth and postpartum based on the recommendations made in the consensus-based standards for the selection of health measurement instruments (COSMIN) and by the International Test Commission (ITC). The study consists of two phases: 1 - Evaluation of the clinimetric properties of the two instruments, which were developed and then altered, based on their comprehensibility and global usability estimated from a pilot study and 2 - Pre-implementation study.

**Discussion:**

The final product will be two valid, reliable, usable instruments for self-assessment of health needs that are highly acceptable to young couples and the professionals who serve them. They will be a valuable resource for meeting the needs of the population more efficiently and guiding decision-making, and they will contribute to the greater sustainability of the health system.

**Supplementary Information:**

**Supplementary information** accompanies this paper at 10.1186/s12884-020-03377-x.

## Background

Becoming a parent is a life experience during which people are especially susceptible to modifying health-related habits [1, 2], which makes health education particularly relevant during this period. It is also a process of profound change, which requires numerous adaptations or restructuring at physical, psychological, social and economic levels [3]. These changes justify the presence of a certain level of anxiety about future challenges, threats and opportunities, as well as the search for guidance and support in Maternal Education (ME).

ME is therefore a complex health intervention, aimed not only at achieving a skillset and knowledge, but also at providing comprehensive support that favors self-management and self-care during pregnancy, childbirth, postpartum and child-rearing [4]. To achieve this, ME must be a dynamic, flexible intervention, constantly updated to adapt to the needs of women and the social contexts of the moment they are living through, and capable of giving a personalized, easily available response when the woman requires it.

In our current social context, pregnant women demand continuous, accessible, rigorous, personalized ME [5]. We also know that they systematically turn to the internet to search for information and advice about their pregnancy, regardless of their social and cultural level [6–8], and that it may even be their main source of information, as in the case of highly qualified women professionals or immigrants, for whom attendance at ME sessions is not always feasible [9, 10].

Given this reality, the use of e-health in ME as a support tool for health decision-making can be useful for women, professionals and the health system [11, 12], as long as it is developed using an appropriate evidence-based methodology. The development of this tool must follow a strategy that considers both the resources and the needs and characteristics of the population which it is aimed at. Therefore, its protagonists (patients, professionals, the healthcare system, the community) should get involved to create a collaborative environment, transfer it to practical use, implement it, and evaluate the process through iterative cycles of continuous improvement [13]. The range of tools currently provided by the internet which respond to these quality requirements is practically non-existent [14].

The processes and components of health decision-making have been extensively studied [15] and all the theoretical models consider the importance of starting with the needs and resources of the patient. However, there are no valid, reliable measurement instruments that address needs during pregnancy, childbirth, postpartum and child-rearing from a comprehensive perspective which adapt to our context. Most of the measurement instruments that can be found in the bibliography aim to evaluate satisfaction, the effectiveness of professional action or the behavior of the population, with the aim of designing future strategies [16–19].

Therefore, an e-health tool to support decision-making by women during the period of pregnancy, childbirth and puerperium should include self-assessment instruments for these needs. Since the range of needs that can arise during motherhood is infinite, it is necessary to prioritize those that are most relevant and require an immediate response. To this end, our research team carried out this prioritization through a Delphi and Nominal Group Technique Approaches study, defining eight topics as the highest priority [20]. In the current project, we intend to respond to two of these priority needs, designing valid, reliable and usable self-applied digital measurement instruments adapted to our context of 1) the needs of women preparing for childbirth and 2) the management of signs and symptoms in the puerperium.

A multidisciplinary group that combines midwives, nurses, pediatricians, epidemiologists, psychologists and pregnant and puerperal women participated in the construction of these instruments. A pool or universe of items was generated from the review and synthesis of the existing bibliography of the available measurement instruments for the different variables that influence coping with childbirth and the puerperium. The content validity and appearance of the test was studied through an iterative process based on the expert participants’ judgment. This group of experts was instructed in the criteria of scientific relevance, clarity, fit to the population and relevance of the scale of response proposed, in order to assess each item. The result was a consensual summary of the items considered relevant, clear and appropriate to the population, with pertinent measurement response options by at least 75% of the participants. This pool of items was transferred to digital format to continue the study.

The comprehensibility and ease of use of these two instruments was evaluated by a pilot test. Ten pregnant women at 37 weeks or more responded to the women’s needs measurement instrument for childbirth and assessed its comprehensibility and ease of use also. A further 10 women in the postpartum period (less than 42 days after delivery) responded to and assessed the comprehensibility and ease of use of the measurement instrument for normal symptoms in postpartum. Recruitment was carried out in the practice of the midwife of reference, who provided: 1. An informed consent form, 2. The link / access to the measurement instrument for the need being studied and 3. Four questions about the comprehension and ease of use of the tool, age and educational level. Any items where comprehensibility and ease of use were considered low by more than 20% of the women were eliminated and the measurement instruments were readjusted based on the comprehensibility of the items and global usability estimated from the pilot study.

The objective of the study is to evaluate the validity, reliability and ease of use of the two self-applied digital instruments for measuring the needs of women in childbirth and identifying alarm symptoms in the puerperium, by analyzing the universality or intergroup variation in the clinimetric parameters.

## Methods/design

### Design

This is a descriptive study of the clinimetric characteristics and usability of two digital instruments for measuring needs in childbirth and postpartum based on the recommendations made in the consensus-based standards for the selection of health measurement instruments (COSMIN) [21] and the International Test Commission (ITC) [22].

### Methods

**Fig. 1:**
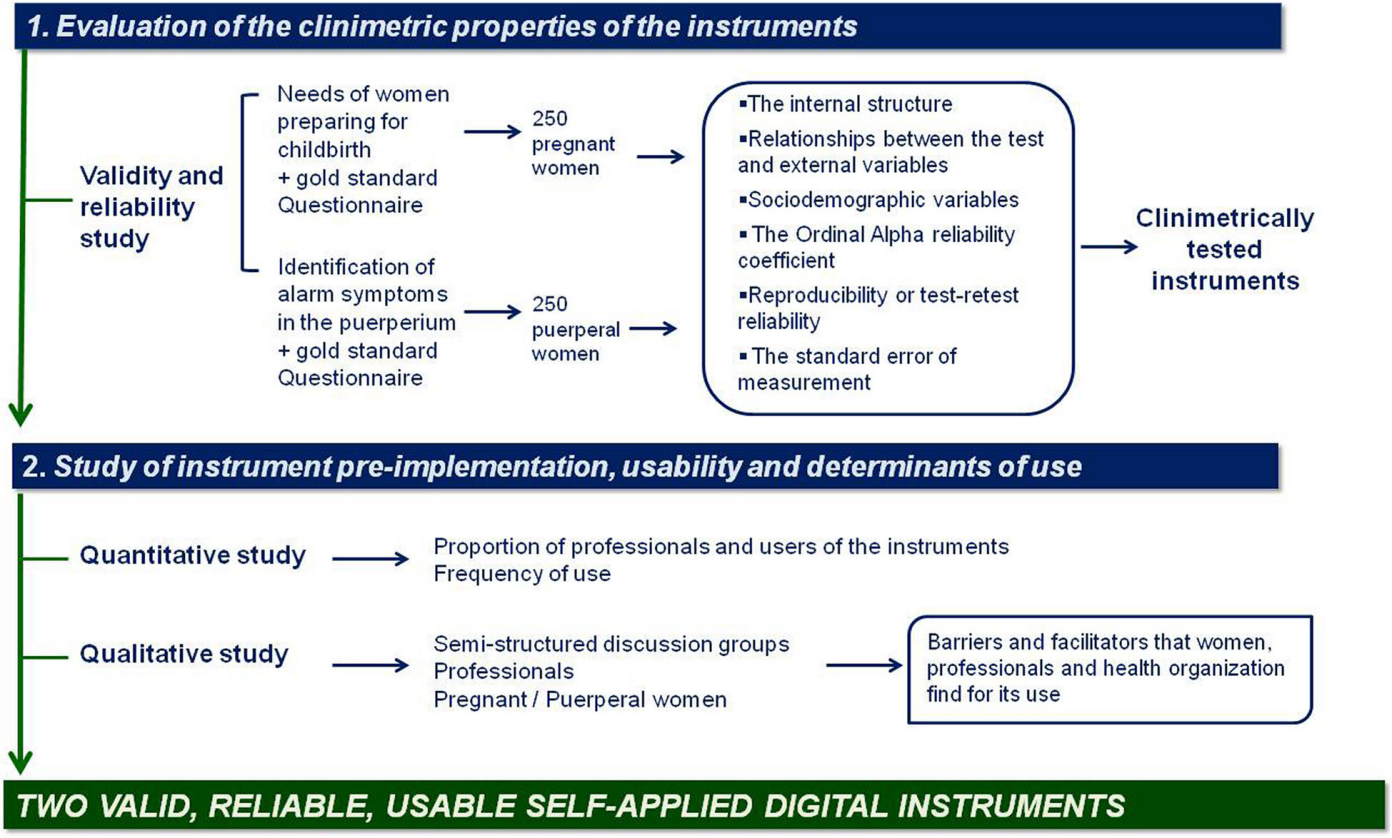
Key milestones in the project’s methodology

The study consists of two phases (Fig. 1):

#### Evaluation of the clinimetric properties of the instruments

##### Participants

All the primary care centers of the Basque Health Service (Osakidetza) in Bizkaia that include among their services the provision of midwifery care for women during pregnancy and the postpartum period. Bizkaia is one of the three provinces that make up the autonomous community of the Basque Country, with a population of 1,142,853 inhabitants [23]. Osakidetza is the public Health Service that provides universal, free care and it is organized in Bizkaia into 5 Integrated Health Organizations with a total of 79 primary care centers and 5 reference hospitals. Of these, 64 primary care centers have a midwife consultation / service and 2 hospitals have a maternity ward.

All the women who come to the midwife’s consultation in the Osakidetza primary care centers in Bizkaia during their pregnancy or during 16 weeks after delivery, who are between 18 and 45 years old, with a current non-pathological pregnancy and without previous pathology, who understand Spanish easily, and consider themselves able to answer the questionnaire in digital format will be eligible for selection.

It is estimated that with a sample of 250 women per instrument, a statistical power of 95% will be available to detect a significant correlation coefficient of 0.20 or greater, both in the case of estimating the test-retest reliability, and to evaluate the concurrent validity with other instruments of measure.

##### Procedure

Each participant will be provided with 1) an informed consent and 2) a link/access to the women’s needs assessment instruments (Additional files 1 and 2), plus the gold standards. This makes it possible to compare the gold standard of the different variables that influence coping with childbirth, such as fear of pain, anxiety, locus of control, self-efficacy and beliefs and knowledge with the dimensions of the new instrument for measuring needs in childbirth (Table 1). And to compare the gold standard of the different variables related to the signs and symptoms during the puerperium, such as self-efficacy, self-image, depression, quality of sleep, breastfeeding self-efficacy, incontinence and sexual satisfaction with the dimensions of the new instrument for measuring needs in postpartum (Table 2). Authorization for the use of gold standard test was requested from the authors.

**Table 1:** Gold Standard of the Needs of women preparing for childbirth instrument

Variables that influence coping with childbirth	Gold Standard
**PHYSICAL STATE**	**Electronic clinical history**
**ANXIETY**	**STAI-AE. Cuestionario de Ansiedad Estado-Rasgo** Spielberger, C. D., Gonzalez-Reigosa, F., Martinez-Urrutia, A., Natalicio, L., & Natalicio, D. S. Development of the Spanish edition of the state-trait anxiety inventory. Interamerican Journal of Psychology. 1971; 5 (3–4), 145–158.
**DEPRESSION**	**Edimburgh potstnatal depresión scale (EDPS)** Garcia-Esteve, L., Ascaso, C., Ojuel, J., & Navarro, P. (2003). Validation of the Edinburgh postnatal depression scale (EPDS) in Spanish mothers. Journal of affective disorders. 2003; *75* (1), 71–76
**ACCEPTANCE OF PREGNANCY QUALITY OF RELATIONSHIP WITH THE PARTNER/PARTNER SUPPORT ATTITUDE TOWARDS CHILDBIRTH**	**Prenatal Self Evaluation Questionnaire.** **Adaptación del cuestionario PSQ (Lederman, 1996)** Armengol Asenjo R, Chamarro Lusar A, García-Dié Muñoz MT. Aspectos psicosociales en la gestación: el Cuestionario de Evaluación Prenatal. Anales de psicología. 2007; vol. 23, n° 1 (junio), 25–32
**FEAR OF PAIN DURING CHILDBIRTH**	**Wijma Delivery Expectancy/Experience Ques-tionnaire (W-DEQ)** Wijma K, Alehagen S, Wijma B. Development of the Delivery Fear Scale. J Psychosom Obstet Gynecol. 2002; 23: 97–107. doi: 10.3109/01674820209042791
**COPING SKILLS**	** Prenatal coping inventory (NuPCI)** Lorén-Guerrero L, Gascón-Catalán A, Romero-Cardiel MA. Adapting the revised prenatal coping inventory (NuPCI) for use in a Spanish population. J Psychosom Obstet Gynaecol. 2018 Jun;39 (2):156–163. doi: 10.1080/0167482X.2017.1315565.
**LOCUS OF CONTROL**	**Multidimensional Health Locus of Control (MHLC)** Tomás-Sábado j. & Montes-Hidalgo J. Versión española de la Escala multidimensional de locus de control de la salud en estudiantes de enfermería. Enfermería Clínica 2016; 26 (3), 181–187.
**CHILDBIRTH SELF-EFFICACY**	**Child Birth Self Efficacy Inventory. CBSEI** Cunqueiro, M. J., Comeche, M. I., & Docampo, D. Childbirth Self-Efficacy Inventory: psychometric testing of the Spanish version. Journal of Advanced Nursing. 2009; 65 (12), 2710–2718.
**BELIEFS ABOUT THE DANGER OF CHILDBIRTH VS NATURAL PROCESS**	**Attitudes towards the Medicalization of Childbirth questionnaire** Benyamini Y, Molcho ML, Dan U, Gozlan M, Preis H. Women’s attitudes towards the medicalization of childbirth and their associations with planned and actual modes of birth. Women Birth. 2017; 30 (5):424–430. doi: 10.1016/j.wombi.2017.03.007.

**Table 2:** Gold Standard for Identification of alarm symptoms in the puerperium instrument

Postpartum signs and symptoms	Gold Standard
**INFORMATION ABOUT THE BIRTH**	**Electronic clinical history**
**URINARY INCONTINENCE**	**ICIQ. International Consultation on Incontinence Questionnaire** Espuña Pons M, Rebollo Álvarez P, Puig Clota M. Validación de la versión española del International Consultation on Incontinence Questionnaire-Short Form. Un cuestionario para evaluar la incontinencia urinaria. Medicina Clínica 2004;122 (8): 288–292
**FUNCTIONAL STATUS**	**SF-12 Health Survey Short Form** Alonso, J., Prieto, L., & Anto, J. M. The Spanish version of the SF-36 Health Survey (the SF-36 health questionnaire): an instrument for measuring clinical results. Medicina clínica. 1995; *104* (20), 771–776.
**SELF CARE**	**PVS. Prescribe vida saludable** Bully, P., Sanchez, A., Grandes, G. et al. Metric properties of the “prescribe healthy life” screening questionnaire to detect healthy behaviors: a cross-sectional pilot study. BMC Public Health**.** 2016; 16**,** 1228 doi: 10.1186/s12889-016-3898-8
**SELF-IMAGE**	**Test EDI-3. Eating Disorder Inventory** Elosua, P., López-Jáuregui, A. y Sánchez-Sánchez, F. Manual de la adaptación al español del Eating Disoder Inventory-3. 2010. Madrid.TEA Ediciones
**DEPRESSION**	**Edimburgh potstnatal depresión scale (EDPS)** Garcia-Esteve, L., Ascaso, C., Ojuel, J., & Navarro, P. (2003). Validation of the Edinburgh postnatal depression scale (EPDS) in Spanish mothers. Journal of affective disorders. 2003; *75* (1), 71–76
**PARENTAL SENSE OF COMPETENCE**	**Test PSOC. Parental Sense of Competence** Oltra-Benavent, P., Cano-Climent, A., Oliver-Roig, A., Cabrero-García, J., & Richart-Martínez, M. Spanish version of the Parenting Sense of Competence scale: Evidence of reliability and validity. Child & Family Social Work.2019. doi: 10.1111/cfs.12693
**SLEEP**	**Test ISI. Insomnia Severity Index** Fernandez-Mendoza, J., Rodriguez-Muñoz, A., Vela-Bueno, A., Olavarrieta-Bernardino, S., Calhoun, S. L., Bixler, E. O., & Vgontzas, A. N. The Spanish version of the Insomnia Severity Index: a confirmatory factor analysis. Sleep medicine. 2012; 13 (2), 207–210.doi: 10.1016/j.sleep.2011.06.019
**SOCIAL SUPPORT**	**Medical Outcomes Study – Social Support Survey. (MOS-SSS**) Gómez-Campelo, P., Pérez-Moreno, E. M., de Burgos-Lunar, C., Bragado-Álvarez, C., Jiménez-García, R., & Salinero-Fort, M. Á. Psychometric properties of the eight-item modified Medical Outcomes Study Social Support Survey based on Spanish outpatients. Quality of Life Research. 2014;23 (7), 2073–2078.
**BREASTFEEDING**	**Prenatal Breast-feeding Self-efficacy Scale** Piñeiro-Albero R M, Ramos-Pichardo J D, Oliver-Roig A, Velandrino-Nicolás A, Richart-Martínez M, García-de-León-González R, Wells K J. The Spanish version of the Prenatal Breast-feeding Self-efficacy Scale: Reliability and validity assessment. International Journal of Nursing Studies. 2013. Volume 50, Issue 10. DOI: 10.1016/j.ijnurstu.2012.12.010
**SEXUAL ACTIVITY/SATISFACTION**	**Escala FSM, Función Sexual de la Mujer** Sánchez F, Pérez Conchillo M, Borrás Valls JJ, Gómez Llorens O, Aznar Vicente J, Caballero Martin de las Mulas A. Diseño y validación del cuestionario de Función Sexual de la Mujer (FSM). Aten Primaria 2004;34 (6):286–94. doi: 10.1157/13067028
**CONTRACEPTION**	**Electronic clinical history**

As one instrument is labor-oriented and the other to detect postpartum alarm symptoms, they will be administered during pregnancy or after childbirth, as appropriate.

##### Measurements

Most of the users will fill out the evaluation instrument along with the gold standards on one occasion and some of them, approximately 50 participants per instrument, will complete it twice, with an interval of approximately 3 weeks (± 1 week) between attempts.

##### Data analysis

The validity study will be carried out by accumulating different types of evidence. The internal structure of the instruments will be evaluated by means of exploratory, confirmatory factor analyses, using the most appropriate estimators in each case and based on the distribution of the scores for the items. The relationships between the test and external variables will include the study of the score association with other tests that measure constructs similar to the instrument. In addition, the differences in the scores obtained in the instrument will be analyzed, based on sociodemographic variables.

The Ordinal Alpha reliability coefficient will be estimated for each subscale and for the secondary scales, thus obtaining an indicator of the internal consistency of the scores and considering the values > 0.70 to be acceptable and > 0.80 to be good. Reproducibility or test-retest reliability will be determined by intraclass correlation. In addition, the standard error of measurement will be calculated, to quantify random errors around the true score.

All analysis will be carried out using the R statistical software package.

#### Study of instrument pre-implementation, usability and determinants of use

##### Participants

Midwives and users from the 64 primary care centers in the health area of ​​Bizkaia within the Basque Health Service (Osakidetza).

##### Procedure

For the study of the tool’s usability and its determinants of use, a mixed methodology will be used:

1. The use of the designed instruments will be described quantitatively, using descriptive statistics of the uptake by the professionals and the women and electronic medical records.
2. A qualitative study will be carried out through semi-structured discussion groups. A semi-structured interview script in relation to the barriers and facilitators of the use of the tools will be developed. Groups of health professionals who have been able to use the instruments will be formed, regardless of how much they have used them, and also groups of women who have been given access to these instruments, regardless of their frequency of use.

Each focus group will be held in a 90-min session moderated by an expert in qualitative research methodologies. All the information will be recorded in audio, it will be transcribed and then a content analysis will be carried out by three team members until saturation is reached.

##### Measurements

The proportion of professionals and users who use the instruments among those likely to do so and the frequency of use in the different centers in the two periods (pregnancy / postpartum) will be evaluated.

The variables that facilitate or hinder the inclusion of these two new instruments in routine professional practice will be identified, by looking at the determinants of this behavior.

Predictive and confounding variables will be analyzed, so the age, nationality, race, social class, educational level, marital status, employment situation, rural or urban environment, distance to hospital, spontaneous pregnancy vs assisted reproduction technique, obstetric history and other clinical characteristics of women will be examined. Furthermore the age, sex, length of time in the position, type of contract and profile of adoption of innovations, measured with Borracci questionnaire [24], of professionals will be examined. Finally, The barriers and facilitators that Integrated Health Organizations and primary care centers find for its use will also be examined, such us number of midwives, rural or urban environment, population served, and organizational predisposition to change measured with the OR4KT questionnaire [25].

##### Data analysis

Frequency analyses will be performed and central trend and dispersion statistics will be used to quantify the use of instruments by the professionals and the women. A usability and feasibility analysis will be made through discussion sessions and interviews. The thematic content analysis method will be used. The researchers will independently read the verbatim transcripts and order the information around the possible barriers or facilitating factors found. This process requires checking the text on multiple occasions, assigning codes to the different segments of the text and later regrouping them into more general or more specific categories. With these, each analyst will subsequently build up a conceptual structure that is pooled (triangulation) in order to contrast it later with the texts and lead to final results.

## Discussion

This project constitutes an essential step in the development of the “modeling, implementation and evaluation of new ME focused on the needs of women” in the multi-method line of other work [26], which will allow us to offer an effective response to real demands of women and their families in the current sociocultural context. These two measurement instruments will be integrated within the framework of this new ME, providing support at the two particularly critical moments of childbirth and postpartum.

Without a doubt, these measurement instruments will help women to identify what their health needs are and the personal resources they have for tackling these stages; they will help them recognize the warning signs and request the necessary help, as well as respond appropriately to normal signs and symptoms, thereby contributing to greater sustainability of the health system.

It is very likely that these instruments in digital format will be well received by the population, since citizens increasingly demand participation in decisions, greater autonomy in their relationship with the health services and greater agility responding to their needs [27, 28]. Furthermore, these instruments have been adapted to the needs of women and comply with the fundamental premise that the end user should participate in their creation [29].

It is thought that they will be well accepted by health professionals, since they are directly involved in their design, preparation and evaluation, thus responding to the limited and necessary participation of health professionals in the integration of care with new technologies [10, 30]. Furthermore, these instruments will provide professionals with valuable resources to meet the needs of the population more efficiently and guide decision-making throughout this stage, contributing to the improvement of prenatal and postnatal care [26] and to a service that is more rewarding for professionals.

We understand that these instruments for self-management of health needs must have a digital support to allow them to be agile and accessible at any time from different devices. Furthermore, the digital nature of the instruments will facilitate health data collection [12], which can guide new interventions and adapt the instruments to the demands of women and their families, since a digital format allows quick changes, flexibility and dynamism.

These instruments will be integrated into a much more complete e-health tool that aims to provide resources to a health system that aims to include the user’s perspective within the care it provides [31]. It is a tool that may be essential in situations of social distancing and restrictions on people’s mobility, such as those we have experienced with the COVID 19 pandemic [32, 33].

The pre-implementation study will make it possible to obtain instruments that, in addition to being valid, are usable and feasible for the women they have been designed for and for healthcare professionals in their daily work. Therefore, we have models such as that proposed by the Medical Research Council, which describe the design of this type of complex intervention, taking into account the variables that make them up, and their development and implementation [34, 35], taking into account the context in which they are to be implanted [36, 37].

In the future, the extension of these two instruments to the rest of the population will be simple, since many health services usually use digital resources in which they can be included. Adaptation to different types of populations will also be feasible, which is especially relevant considering that some population groups hardly access any of the current educational activities.

In terms of the study’s limitations, it could be seen as a sample that will be selected in a non-random way, although we do not see its representativeness as compromised, given the participation of several health centers with different socioeconomic levels, the consecutive recruitment of women, and privacy for each woman to answer the questionnaire. Another possible limitation, which is frequent in clinical studies, is the difficulty of uptake due to tiredness or lack of time among the professionals who have to implement it. In our study, the involvement of these professionals right from the design stage would facilitate gathering the necessary sample.

The final product will be two valid, reliable and usable instruments for self-assessment of health needs, which will be highly accepted by young couples and by the professionals who care for them and which will contribute to the greater sustainability of the health system.

## Supplementary Information

**Additional file 1.** Needs of women preparing for childbirth instrument.

**Additional file 2.** Identification of alarm symptoms in the puerperium instrument.

## Data Availability

The study is ongoing. We have not completed participant recruitment for the evaluation of the clinimetric properties of the instruments. Data will be available when the study ends (December 31, 2021).

## Abbreviation

ME: Maternal Education
