# Supplementary material for: Development of two instruments for assessing maternity health needs: protocol of a clinimetric study
Source: BMC Pregnancy Childbirth. 2020 Nov 17;20:701. doi: 10.1186/s12884-020-03377-x (PMC7670976; doi:10.1186/s12884-020-03377-x)
Supplement: Supplementary file 1 — Additional file 1. Needs of women preparing for childbirth instrument. [file 12884_2020_3377_MOESM1_ESM.pdf]

Needs of women preparing for childbirth instrument

This questionnaire is part of a STUDY entitled "Development and analysis of the metric properties of two instruments for assessing health needs before childbirth and during puerperium", which is being carried out by midwives in the Basque Health Service (OSAKIDETZA) for the creation of a measurement instrument for these women's needs that is valid, reliable and appropriate for our area. The study, led by Isabel Artieta and Carmen Paz, midwives in the Zuazo and Sestao health centers respectively, is funded by the Basque Government Department of Health and has been approved by the Euskadi Clinical Research Ethics Committee (CEIC Euskadi).

Your participation will consist of COMPLETING THIS QUESTIONNAIRE, which presents a list of questions related to the expectations, feelings or preferences that different women may have during pregnancy and childbirth. You only have to indicate your degree of agreement with each of them, ticking the box of the alternative that is closest to what you think. The questionnaire will take around 45 MINUTES and will allow you to reflect on what type of delivery may be most suitable for you and which may be accessible in your environment.

This study is not for profit. Participation is voluntary and you can withdraw from the study at any time you want and for whatever reason you may have.

ALL YOUR DATA will be treated according to CURRENT LEGISLATION (EU Regulation 2016/679 of the European Parliament and of the Council of April 27, 2016 regarding the protection of natural persons with regard to the processing of personal data and free movement of this data and to Organic Law 3/2018, of December 5, on the Protection of Personal Data and guarantee of digital rights that repeals Organic Law 15/1999, of December 5, on the protection of personal data). Personal data will be processed by Osakidetza – Basque Health Service. NO DATA WILL BE TRANSFERRED TO THIRD PARTIES, except under legal obligation. If necessary, we may have to contact you on occasion in relation to this study. You have the right of access, rectification, deletion of your data, and the limitation or opposition to its treatment. For further information on data protection, see the following website:

http://www.osakidetza.euskadi.eus/protecciondatos

You can ANSWER on any MOBILE PHONE, TABLET OR LAPTOP. You can ANSWER OVER VARIOUS SESSIONS without losing information by using the option I WILL CONTINUE LATER.

1. I have read and understood the information and I agree to participate voluntarily
    - ✓ I accept

2. Please enter your health card number (TIS).
    TIS number:

3. How old are you?

4. What is the maximum level of studies you have completed?
    - o Without studies
    - o Primary studies
    - o Secondary studies to 16
    - o Sixth form studies / Baccalaureate
    - o Diploma / Bachelor's Degree / University Degree
    - o PhD

5. What is your employment situation?

- I am a student
- I am employed
- I am a housewife
- I am unemployed
- I have a disability

  Other (Please specify)

6. What is the occupation of the person with the highest-paid employment situation in the family?

7. The person with the best employment situation in the family...

- is self-employed without employees
- is self-employed in a company with less than 10 employees
- is self-employed in a company with 10 or more employees
- is the Manager of a company with less than 10 employees
- is the Manager of a company with 10 or more employees
- is an overseer, supervisor or manager
- Other wage earner

8. What is your country of origin?

  Spain

  Other (Please specify)

9. What week of pregnancy are you in at the moment?

10. How much do you currently weigh? In kilos

11. How tall are you? In meters

12. Below is a series of questions related to your health.
Answer **YES** or **NO** by checking the corresponding box.

- Will you be over 40 when the baby is born?
- Before becoming pregnant, did a healthcare worker tell you that you were overweight, or that you were excessively thin?
- Do you have any illness that requires regular medical control?
- In your family or that of the baby’s father, is there anyone with a birth defect diagnosed as genetic or inherited (e.g. spina bifida, heart defect or Down syndrome)?
- Have you had a previous child born before 37 weeks of pregnancy?
- Have you had a previous child born with low weight (<2500gr) or high weight (> 4000g)?
- Have you had a previous pregnancy that resulted in fetal death between 22 weeks of pregnancy and 7 days postpartum?
- Have you had any complications in this pregnancy that require monitoring as a high-risk pregnancy, such as gestational diabetes, placenta previa or pre-eclampsia?
- Have you consumed alcohol during this pregnancy?
- Have you smoked during this pregnancy?
- Have you taken any illegal drugs during this pregnancy?
- During this pregnancy, have you taken a vitamin supplement with folic acid and/or iodine?
- Have you taken any medication on a regular basis?

13. During the LAST TWO WEEKS, how often have you felt the following?
Never / Almost never / Sometimes / Often / Always
- I have felt anxious
- I have felt that I could not stop or control my worries
- I have had little desire to do things
- I have felt down, depressed or desperate

14. Have any of the feelings you mentioned earlier made it difficult for you to carry out your normal activities?
- YES
- NO

15. How much do you agree with each of these statements?
Strongly disagree / Disagree / Neutral / Agree / Strongly agree

- I am very happy to be pregnant
- This is a planned pregnancy
- So far, it has been a difficult pregnancy
- I had a hard time accepting this pregnancy

16. Do you have a partner at the moment?
- YES
- NO

17. How much do you agree with each of these statements?
Strongly disagree / Disagree / Neutral / Agree / Strongly agree
- I have the support of my partner when I feel overwhelmed
- I consider that as a couple we have a good level of communication
- I think that I will be able to count on the support of my partner during the delivery
- I can count on my partner for the care I need during pregnancy
- I think I can count on my partner to take care of the baby
- My partner and I talk about pregnancy whenever I need to

18. Regarding this series of statements, how often would you say each of them have occurred?
Never / Almost never / Sometimes / Often / Always
- I ask doctors or midwives about the delivery
- I think about what things will be like after the baby arrives
- I plan how I am going to act during the delivery
- I spend time or talk with people who have just had a baby
- I imagine what the delivery will be like
- I spend time with or talk to other pregnant women
- I talk with family or friends about what it's like to give birth
- I look for information in books and on the internet about pregnancy, childbirth, etc.

19. How much do you agree with each of the following statements?
Strongly disagree / Disagree / Neutral / Agree / Strongly agree
- To be calmer, I try not to think about the delivery
- Thinking about childbirth makes me so upset that I wish it was over now
- Thinking about childbirth makes me feel so upset that I wish I wasn't pregnant

20. How strongly do you agree with each of the following statements?
Strongly disagree / Disagree / Neutral / Agree / Strongly agree
- I am sure my experience of giving birth will depend on my applying everything I know
- I am more likely to have the kind of experience I want if I plan the delivery
- My delivery will depend mainly on the gynecologist or midwife who assists me

- The hospital rules will be the greatest reason behind whether I have, or not, the type of delivery I want
- I can largely determine the progress of my labor
- Knowing my rights, I will be able to protect my interests during delivery
- Whether or not I have a complication will depend on how my gynecologist and midwife take care of my pregnancy
- My delivery will depend on my ability to follow the instructions of the gynecologist or midwife faithfully
- My delivery will be determined by my own actions.
- Suppose you are in labor, during the dilation stage; imagine how you will feel and try to see yourself with contractions every five minutes or less.

21. Indicate your degree of agreement regarding the following questions:

Strongly disagree / Disagree / Neutral / Agree / Strongly agree

- I believe that I will be able to push enough during labor for my baby to be born
- I will cope well during delivery, as with other challenges in my life
- I think I will be able to stay calm
- I will be able to focus on helping the delivery, even if other things happen around me
- I think I will feel comfortable giving birth in the presence of the medical team
- The delivery will be a satisfying experience for me
- I can cope with the idea that labor might take a long time
- I think I will be able to detect the moment of labor onset
- I think I will be able to go to the hospital at the right time
- I think my body is perfectly prepared to give birth
- I am able to bear the pain

22. How much do you agree with each of the following statements?

Strongly disagree / Disagree / Neutral / Agree / Strongly agree

- Childbirth requires vigilant medical supervision.
- There are many things that can go wrong during labor.
- Childbirth is a medical process.
- Childbirth is a dangerous process.
- Today, there is no reason for women to experience labor pain.
- Childbirth is an empowering experience.
- Pain during labor is an important/significant part of the experience.
- Childbirth is a natural process
- A woman's body knows how to react at the time of delivery.
- Dilation/labor should be allowed to progress at its own pace

23. Indicate in the following points your degree of fear of childbirth
    - Not at all
    - A lot

24. Indicate your degree of anxiety or nervousness before the birth
    - Not at all
    - A lot

25. Indicate your degree of fear of childbirth due to bad experiences of your own or those you know
    - Not at all
    - A lot

26. Indicate your degree of fear of complications for your health arising from childbirth
    - Not at all

- A lot

27. Indicate your degree of fear, right now, that the baby will suffer during delivery
    - Not at all
    - A lot

28. Regarding the following preferences, how much do you agree with each of them?

Strongly disagree / Disagree / Neutral / Agree / Strongly agree

- Regarding the center, for me it is very important that it has high technology. My priority is that any unforeseen event related to my health or that of my child can be resolved as quickly as possible.
- For me it is very important to have an individual room
- I appreciate that in the center, medical intervention will only be for emergencies. My priority is a natural delivery whenever possible.
- What I value is the privacy of my home. I prefer to spend the dilation period at home attended by a midwife who is an expert in home birth
- For me it is important that the professionals who are going to attend me introduce themselves and inform me about their training/functions.
- I attach importance to being treated by the same people during my time in dilation and delivery
- It would be important to be accompanied by the same professionals who have attended me during my pregnancy (gynecologist, midwife)
- It is important that a health professional or doula accompany me as long as possible during dilation
- The more professionals who attend me (gynecologists, midwives, pediatricians), the more secure I will feel.
- I would like to be accompanied by my partner or a person I trust throughout the period
- I would like to be accompanied by the most significant people for me at that moment: partner, family, other children, friends. ..
- For me it is important to be able to decide at all times who I want to accompany me
- I would like to wear the clothes that the hospital provides
- I would like to have an atmosphere that is as intimate as possible
- Even if I am exposed to the presence of professionals in training, I would like to give birth in a public center

Below are a series of statements followed by three options to choose from. Mark the one that best corresponds to your preferences during the dilation period

29. For the environment I prefer...
    - I prefer a homely, cozy, dimly lit, quiet environment ...
    - I feel safer in a hospital environment, with high technology in case any setback arises
    - I have no preference, the atmosphere and decoration is the least of it

30. Regarding the position I prefer...
    - I would like to move, change posture, and have the option of using a ball, cushions, ropes, or other supportive methods that facilitate the progression of labor
    - I prefer to be lying in bed, as comfortable as possible
    - I have no preferences, whatever they tell me

31. During the period of dilation
    - I would like to be able to eat or drink if I need it at that moment without any kind of restriction.
    - I would like to be able to drink during the dilation period if I am thirsty.

- Regarding the intake of liquids, I do not have any preference; the staff who attend me at all times will indicate whether or not I can drink

32. Regarding your preferences about methods to relieve pain and about possible interventions during the dilation period, indicate your degree of agreement with the following statements
Strongly disagree / Disagree / Neutral / Agree / Strongly agree

- I would prefer to use non-pharmacological methods to relieve pain during the period of dilation
- I would like to use epidural analgesia
- I would like to use some type of analgesia that I know (hot tub, local heat, nitrous oxide, lumbar massage, sterile water injections, tens…)
- I would like to be able to use some method to calm the pain that I can use according to my needs
- I assume the performance of medical operations that the doctors may see necessary.
- I want to be informed of all the medical operations that are going to be carried out and my consent is requested before they are carried out
- I prefer not to have my waters broken artificially unless it is strictly necessary and always with my consent.
- If the administration of oxytocin is indicated, I want to be informed and to have my consent requested
- I would approve of having the strictly necessary vaginal examinations performed and always with my consent
- Although it may limit my movement, I prefer continuous fetal monitoring because I think it is safer for the baby
- Having an intravenous drip gives me security and does not reduce my comfort
- If I have difficulty urinating spontaneously, I would like to be informed and asked for my consent before being put on a urinary catheter.
- Provided that I am previously informed and my consent is requested, I have no problem in having any procedure that is not strictly necessary, if by doing so I am collaborating in the training of professionals, as long as they do not pose a risk to me or my baby
- I want a spontaneous onset of labor and would like to resort to labor induction only with a clear medical indication

Below are a series of statements followed by different options to choose from. Indicate the one that best corresponds to your preferences during the stage of expulsion and delivery

33. Regarding the environment that I prefer
    - I would like to give birth in a homely, cozy, dimly lit, quiet environment ...
    - I would like to give birth in a hospitable environment, with high technology in case any setback arises
    - I have no preference, the atmosphere and decoration is the least of it

34. As for the position I prefer ...
    - I would like to have the freedom to adopt the posture that is most comfortable for me during the expulsion: squatting, on all fours, in a gynecological position, or giving birth in a bath of warm water, if possible.
    - As I intend to give birth with epidural anesthesia, I understand that this type of anesthesia may somehow condition my posture during delivery
    - I have no preference in this regard, I will adopt the position indicated by the professionals

35. How much do you agree with the following statements?

Strongly disagree / Disagree / Neutral / Agree / Strongly agree

- I would like to be allowed, at the moment of expulsion, to push when I feel the need
- I would like to be able to see the baby coming out in a mirror
- I would like to be able to touch the baby's head when it appears
- I would like to be able to extract the baby
- As long as there is no risk to the baby or to me, I prefer not to have an episiotomy, even if it takes longer.
- I wish to be informed and request my consent for any procedure: episiotomy, instrumental delivery (forceps, suction cup, spatulas) or caesarean section
- I want the caesarean section to be a last resort, when no other possibility is seen
- I prefer a spontaneous delivery (delivery of the placenta), without any intervention
- I prefer active management of the delivery, with the help of a drug, if this reduces the possibility of postpartum hemorrhage
- It is important for me to wait for the umbilical cord to stop pulsing before clamping it.
- For me it is important to initiate skin-to-skin contact immediately after the delivery or caesarean section
- For me it is important that the first check-ups of the baby are carried out in skin-to-skin contact
- For me it is important to be able to start breastfeeding the baby in the first two hours of life
- I want the baby to receive all the usual preventive treatments and interventions
- It is important for me to stay with my baby at all times
- I would like to feed my baby whenever I think she/he asks for it
- For me it is important that the baby does not receive formula milk unless it is essential

Thank you very much for your cooperation
