# Supplementary material for: Development of two instruments for assessing maternity health needs: protocol of a clinimetric study
Source: BMC Pregnancy Childbirth. 2020 Nov 17;20:701. doi: 10.1186/s12884-020-03377-x (PMC7670976; doi:10.1186/s12884-020-03377-x)
Supplement: Supplementary file 2 — Additional file 2. Identification of alarm symptoms in the puerperium instrument. [file 12884_2020_3377_MOESM2_ESM.pdf]

Identification of alarm symptoms in the puerperium instrument.

This questionnaire is part of a STUDY entitled “Development and analysis of the metric properties of two instruments for assessing health needs before childbirth and during puerperium”, which is being carried out by midwives in the Basque Health Service (OSAKIDETZA) for the creation of a measurement instrument for these women’s needs that is valid, reliable and appropriate for our area. The study, led by Isabel Artieta and Carmen Paz, midwives in the Zuazo and Sestao health centers respectively, is funded by the Basque Government Department of Health and has been approved by the Euskadi Clinical Research Ethics Committee (CEIC Euskadi).

Your participation will consist of COMPLETING THIS QUESTIONNAIRE, which presents a list of questions related to the signs and symptoms that are normal in the postpartum period. You only have to indicate your degree of agreement with each of them, ticking the box of the alternative that is closest to what you think. The questionnaire will take around 30 MINUTES.

This study is not for profit. Participation is voluntary and you can withdraw from the study at any time you want and for whatever reason you may have.

ALL YOUR DATA will be treated according to CURRENT LEGISLATION (EU Regulation 2016/679 of the European Parliament and of the Council of April 27, 2016 regarding the protection of natural persons with regard to the processing of personal data and free movement of this data and to Organic Law 3/2018, of December 5, on the Protection of Personal Data and guarantee of digital rights that repeals Organic Law 15/1999, of December 5, on the protection of personal data). Personal data will be processed by Osakidetza – Basque Health Service. NO DATA WILL BE TRANSFERRED TO THIRD PARTIES, except under legal obligation. If necessary, we may have to contact you on occasion in relation to this study. You have the right of access, rectification, deletion of your data, and the limitation or opposition to its treatment. For further information on data protection, see the following website:

http://www.osakidetza.euskadi.eus/protecciondatos

YOU CAN ANSWER ON YOUR MOBILE PHONE, and if so, to be able to view it correctly you must hold the screen IN A HORIZONTAL POSITION.

1. I have read and understood the information and I agree to participate voluntarily

   ✓ I accept

2. Please enter your health card number (TIS).

   TIS number:

3. How old are you?

4. What is the maximum level of studies you have completed?
   - Without studies
   - Primary studies
   - Secondary studies to 16
   - Sixth form studies / Baccalaureate
   - Diploma / Bachelor's Degree / University Degree
   - PhD

5. What is your employment situation?
- I am a student
- I am employed
- I am a housewife
- I am unemployed
- I have a disability

Other (Please specify)

6. What is the occupation of the person with the highest-paid employment situation in the family?

7. The person with the best employment situation in the family...
- is self-employed without employees
- is self-employed in a company with less than 10 employees
- is self-employed in a company with 10 or more employees
- is the Manager of a company with less than 10 employees
- is the Manager of a company with 10 or more employees
- is an overseer, supervisor or manager
- Other wage earner

8. What is your country of origin?

Spain

Other (Please specify)

9. Due date

10. How many babies were born in this delivery?

11. How much did the baby weigh (if more than one, indicate the heaviest)? In grams

12. What kind of delivery did you have?
- Normal vaginal delivery
- Vaginal delivery with the help of a vacuum cup, forceps or spatulas
- Vaginal breech delivery
- Caesarean section
- Others (for example twin with 1 normal delivery + 1 forceps etc...)

13. Did you have epidural anesthesia?
- Yes
- No

14. Did you have any complications during delivery?
- No
- Yes, fever during labor
- Yes, high blood pressure
- Yes, postpartum hemorrhaging that required a blood transfusion, and/or admission to the ICU
- Yes, manual removal of the placenta
- Yes, complications other than the above

15. Did you have an episiotomy (cut to facilitate the delivery of the baby)?
- No
- Yes

16. Did you have any tearing during delivery?

- No
- Yes

17. Have you had any problem in the genital area (hematoma, inflammation...)?
    - No
    - Yes

18. At the moment, do you have hemorrhoids?
    - No
    - Yes

19. Was it your first pregnancy?
    - No
    - Yes

20. If it is not the first, how many pregnancies have you had before this one?

21. Do you have pain in any part of your body?
    - Yes
    - No

If YES: Assess your degree of pain in the following areas:
0-1-2 (No pain) / 3-4 / 5-6 / 7-8 / 9-10 (A lot of pain)

- The genital and/or anal area
- The abdominal area (belly)
- One or both breasts
- One or both nipples
- Head
- One leg, accompanied by swelling, warmth, and local redness
- Other areas of the body

22. Do you have any of these symptoms?

Answer **YES** or **NO** by checking the corresponding box.

- Vaginal bleeding heavier than a period
- Foul-smelling vaginal discharge/secretion
- Temperature higher than 38ºC (fever) in the last 12 hours
- Temperature higher than 38ºC (fever) now
- Feeling of extreme tiredness
- Dizziness or fainting fits at any time after the delivery
- One or both breasts hard or swollen even after breastfeeding
- Any red spots on the breast
- Injury to one or both nipples
- Difficulties, flashing lights or other sudden disturbances in vision
- Constipation

24. During the last 4 weeks my physical condition has limited me...

Never / Rarely / Sometimes / Often / Daily

- In carrying out basic baby-care tasks (breastfeeding, bathing, changing diapers, etc.)
- In making moderate efforts such as moving a table, sweeping or scrubbing the house, walking for more than an hour ...

34. Are you happy with your current physical appearance?
    - Yes
    - No

35. Rate how often you have the following thoughts
Never / Rarely / Sometimes / Often / Always

- I avoid situations in which people can see my body (pool/beach, bathrooms, changing rooms...)
- I am worried about being fat
- I am afraid that my breasts will lose their shape or firmness
- Seeing my body in the mirror makes me feel bad.
- I think I should go on a diet.
- I think I have lost most of the kilos that I gained during pregnancy.
- I think my appearance is normal for a woman who has recently given birth

37. During the past 15 days, how often has the following happened to you?
Never / Rarely / Sometimes / Often / Always

- I have had trouble sleeping
- I have felt alone
- I have cried a lot for no reason
- I have not been able to concentrate on anything
- It seemed to me that I was not myself
- I have felt a failure as a mother
- I have begun to think that I would better off dead

48. Rate how often the following happens to you:
Never / Rarely / Sometimes / Often / Always

- I am able to keep my baby entertained
- I am able to feed my baby
- I am able to bathe my baby
- I can calm my baby when she/he is crying
- I am able to calm my baby when she/he is anxious
- I can calm my baby when she/he cries continuously
- I know when my baby is tired and needs to sleep.
- I am able to understand what my baby wants
- I think my baby responds well to me when I talk to her/him, when I smile, ...
- I think my baby and I have a good interaction with each other
- I can show affection to my baby.

50. Are you satisfied with your quality of sleep?

- Yes
- No

If YES: How often does the following happen to you?
Never / Rarely / Sometimes / Often / Daily

- I wake up in the middle of the night.
- I have sleep problems due to night-time childcare.
- I have trouble sleeping due to baby-related distress
- I have sleep problems that make me feel I have no energy during the day
- I have difficulty falling asleep.
- I have sleep problems due to postpartum physical conditions

51. How often do the following situations occur:
Never / Rarely / Sometimes / Often / Always

- There is a person who is around when I am in a difficult situation
- There is a special person I can share joys and sorrows with
- My family really tries to help me

- I get the help and emotional support I need from my family
- I can count on my friends when things go wrong
- I can talk about my problems with my family
- I have friends I can share my sorrows and joys with
- There is a special person in my life who cares about my feelings
- My family is willing to help me make a decision

54. Are you breastfeeding your baby?
  - Yes
  - No

If YES:
What is your degree of agreement with the following statements?
Strongly disagree / Disagree / Neither agree nor disagree / Agree / Strongly agree
- The benefits of breast milk last, even after the baby has been weaned
- Breastfeeding increases the mother-child bond.
- Bottle-fed babies tend to receive more food than breast-fed babies
- Breast-fed babies grow up healthier than formula-fed babies.
- Breast-fed babies tend to receive more food than bottle-fed babies
- Breast milk is the ideal food for babies.
- Breast milk is easier to digest than artificial milk.
- Breast milk is better than artificial milk.
- A mother who occasionally drinks alcohol should not breastfeed her baby.

Rate the frequency of the following questions
Never / Rarely / Sometimes / Often / Always
- I plan to continue breastfeeding my baby for the next few months
- I have offered my baby formula milk before 4 months of age
- I have given my baby other types of food (fruits, vegetables, cereals) before 4 months
- I have thought about stopping breastfeeding my baby
- I have had difficulties with breastfeeding due to my work
- I have had difficulties with breastfeeding due to family problems
- I have had difficulties with breastfeeding due to too little milk
- I have had difficulties with breastfeeding because the child does not accept the milk
- I have had difficulties with breastfeeding due to nipple problems
- My partner and family motivate and support me to continue breastfeeding
- Feeling good and satisfied motivates me to continue breastfeeding
- Keeping the baby healthy is a motivation to continue breastfeeding
- I get comfortable to breastfeed my baby
- I look for a good position to breastfeed my baby

How often does the following happen to you?
Never / Rarely / Sometimes / Often / Always
- I am able to know if my baby is getting enough milk at each feed
- I can breastfeed my baby without using formula or powdered milk as a supplement
- I am sure that my baby latches on well to the breast during feeding
- I can handle breastfeeding to my satisfaction.
- Breastfeeding is a satisfying experience for me
- I can breastfeed my baby on one breast and, when finished, switch to the other.

55. Have you resumed sexual activity (alone or with a partner)?
  - Yes
  - No

If YES:
Rate the frequency of the following questions
Never / Rarely / Sometimes / Often / Always

- I have felt arousal during sexual activity
- I have noticed my vagina is lubricated or wet during sexual activity
- I have felt pain or discomfort when touching or caressing the vulva and perineum, during sexual stimulation
- I have felt pain or discomfort at the time of vaginal penetration
- I have felt pain or discomfort during vaginal penetration
- I have felt pain or discomfort after vaginal penetration

If you have felt pain or discomfort when touching or caressing the genital area, how would you classify the intensity of the pain or discomfort?
0-1-2 (No pain) / 3-4 / 5-6 / 7-8 / 9-10 (A lot of pain)

In the last 4 weeks, assess your degree of satisfaction with:
Not at all / A little / Some / Quite a bit / Totally

- Sexual desire or interest
- The intensity of sexual arousal
- The quality of your orgasms
- Your disinhibition and surrender to sexual pleasure during sexual intercourse
- Your concentration during sexual activity
- The ease of lubricating (getting wet) during sexual activity
- How your partner takes into account your sexual needs.

56. Rate your agreement with the following statements
Strongly disagree / Disagree / Neither agree nor disagree / Agree / Strongly agree

- I consider that I am at risk of becoming pregnant at the moment
- It is necessary to use some type of contraceptive method
- I know all the options I have to avoid pregnancy at the moment

Thank you very much for your cooperation
